# Effect of high-pressure torsion on microstructure, mechanical properties and corrosion resistance of cast pure Mg

**DOI:** 10.1007/s10853-018-2779-1

**Published:** 2018-08-13

**Authors:** Donya Ahmadkhaniha, Yi Huang, Matias Jaskari, Antti Järvenpää, Mahmoud Heydarzadeh Sohi, Caterina Zanella, L. Pentti Karjalainen, Terence G. Langdon

**Affiliations:** 1 Department of Material and Manufacturing, School of Engineering, Jönköping University, P.O. Box 1026, Gjuterigatan 5, SE-551 11 Jönköping, Sweden; 2 Materials Research Group, Department of Mechanical Engineering, University of Southampton, Southampton, SO17 1BJ UK; 3 Department of Design and Engineering, Faculty of Science and Technology, Bournemouth University, Poole, Dorset BH12 5BB UK; 4 Kerttu Saalasti Institute, University of Oulu, Pajatie 5, 85500 Nivala, Finland; 5 School of Metallurgy and Materials, College of Engineering, University of Tehran, PO Box 1155-4563, Tehran, Iran; 6 Centre for Advanced Steels Research, University of Oulu, PO Box 4200, 90014 Oulu, Finland

## Abstract

High-pressure torsion (HPT) processing was applied to cast pure magnesium, and the effects of the deformation on the microstructure, hardness, tensile properties and corrosion resistance were evaluated. The microstructures of the processed samples were examined by electron backscatter diffraction, and the mechanical properties were determined by Vickers hardness and tensile testing. The corrosion resistance was studied using electrochemical impedance spectroscopy in a 3.5% NaCl solution. The results show that HPT processing effectively refines the grain size of Mg from millimeters in the cast structure to a few micrometers after processing and also creates a basal texture on the surface. It was found that one or five turns of HPT produced no significant difference in the grain size of the processed Mg and the hardness was a maximum after one turn due to recovery in some grains. Measurements showed that the yield strength of the cast Mg increased by about seven times whereas the corrosion resistance was not significantly affected by the HPT processing.

## Introduction

In recent years, magnesium and its alloys have emerged as candidate biodegradable materials in cardiovascular and musculoskeletal applications [1, 2]. However, the promising biodegradable applications of Mg and its alloys depend on the ability to control their mechanical strength and the corrosion rate in body fluids. Therefore, much research has focused on the special processing of Mg and its alloys in order to modify the properties and thereby commercialize biodegradable Mg alloys. Several methods have been proposed to protect Mg against corrosion, such as Mg purification [3], the use of coatings [4–6] and alloying with other elements [7–9]. Since Mg in the as-cast condition suffers from both low mechanical strength and poor corrosion resistance, it is challenging in practice to find a processing route which can enhance both properties simultaneously.

It is known that mechanical processing can increase the mechanical strength via grain refinement and modify the texture of materials to thereby have an impact on the corrosion behavior. Furthermore, different techniques have been applied, such as extrusion [10], surface mechanical attrition treatment and equal-channel angular pressing (ECAP) [11], to improve the strength and corrosion resistance through the refined microstructure.

Although these processing procedures are effective for achieving significant grain refinement, several experiments have suggested that the use of high-pressure torsion (HPT) may have some benefits because of the ability to refine the microstructure more effectively and also to process alloys with low deformability at room temperature [12–14]. Processing by HPT is a severe plastic deformation (SPD) technique that applies high pressure and shear strain to a thin disk or ring held between two rotating anvils [15]. In the present investigation, HPT processing was applied to cast pure Mg disk samples and mechanical testing and the corrosion performance were evaluated both before and after the HPT. By choosing pure Mg as the workpiece, it was possible to evaluate the effect of microstructure on the corrosion resistance without interference from the distribution and size of any intermetallic and secondary phases.

## Experimental material and procedures

Commercially pure as-cast Mg disks, having diameters of 10 mm and thicknesses of 0.8 mm, were processed by HPT by compressing and then deforming between two anvils under an applied pressure of 6.0 GPa at room temperature to totals of either one or five complete turns. This processing was conducted under quasi-constrained conditions in which there is a small outflow of material around the periphery of the disk during the processing operation [16, 17]. Care was taken to ensure there was no slippage during HPT by placing marker lines on the upper and lower surfaces of each disk prior to processing [18].

The microstructures of the samples, both at the top surfaces and at the cross sections, were characterized using a scanning electron microscope (SEM; Zeiss Ultra Plus) equipped with an electron backscatter diffraction (EBSD) device (Zeiss-Oxford Instruments, 15–20 kV, aperture 120 μm, working distance 10–12 mm, step size typically < 0.6 µm; HKL Channel5 software). The as-cast Mg and HPT-processed samples were cold mounted and a very gentle grinding was used before the polishing procedure to avoid introducing any deformation layer. The incomplete pole figures (0001, 10$\bar{1}$0, 11$\bar{2}$0) were used to determine the texture. Areas having a minimum size of 100 × 100 µm^2^ were scanned in order to include an adequate number of grains in the texture analyses for all samples except in the initial cast structure. The grain size was measured from the EBSD data using the equivalent circular diameter in the HKL Channel5 software.

**Figure 1:**
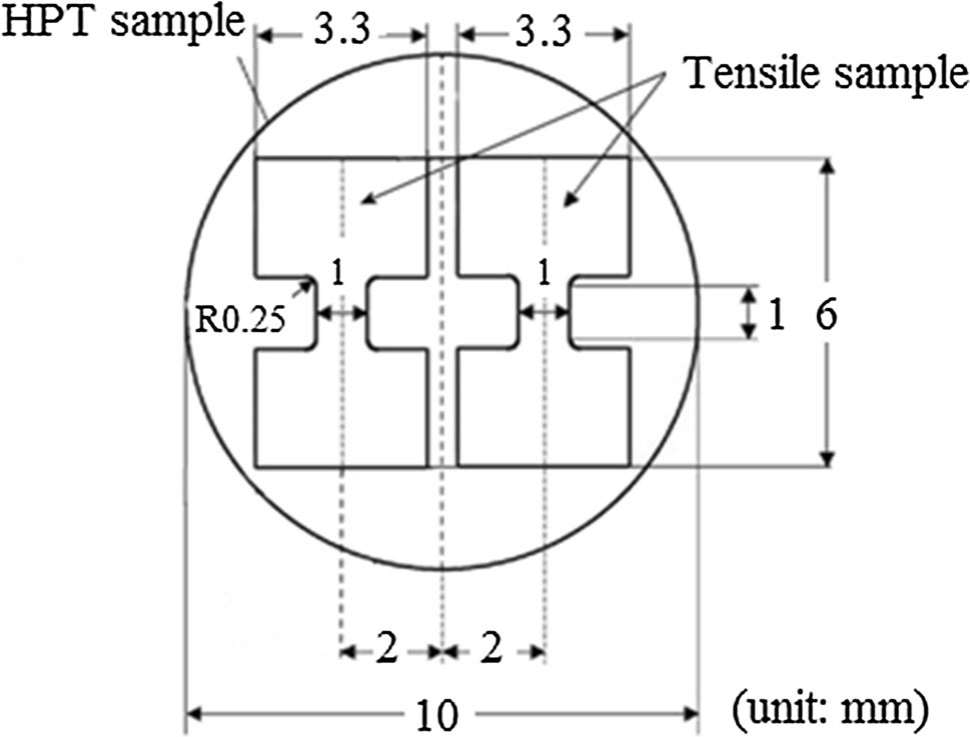
Dimensions of mini-tensile samples cut from HPT disks [19]

Microhardness measurements were carried out on the surfaces of samples along randomly selected diameters using a nano-test Vantage apparatus with a Vickers indenter (load 100 mN) and a dwell time of 10 s. A Zwick Z030 testing machine was used to investigate the tensile behavior at room temperature of miniature tensile samples cut from the HPT disks. Two tensile samples were prepared from off-center positions for the initial as-cast Mg and the HPT-processed disks as shown in Fig. 1 [19], and these samples were tested using initial strain rates of 1.0 × 10^−3^ s^−1^.

A horizontal three-electrode electrochemical cell, with platinum as a counter-electrode, Ag/AgCl (3 M KCl, 0.21 vs. SHE/V) as a reference electrode and Mg sample as a working electrode, was chosen to evaluate the corrosion resistance in a 3.5% NaCl solution. The sample surfaces were ground with SiC abrasive papers up to 1000 grade before initiating the electrochemical testing. Electrochemical impedance spectroscopy (EIS) was carried out at an open-circuit potential (OCP) with an initial delay of 10 min by applying a sinusoidal wave of 10 mV amplitude over a frequency range from 10 mHz to 100 kHz. A ZSimpwin commercial software was used to fit the experimental data.

## Experimental results

### Microstructure and texture

**Figure 2:**
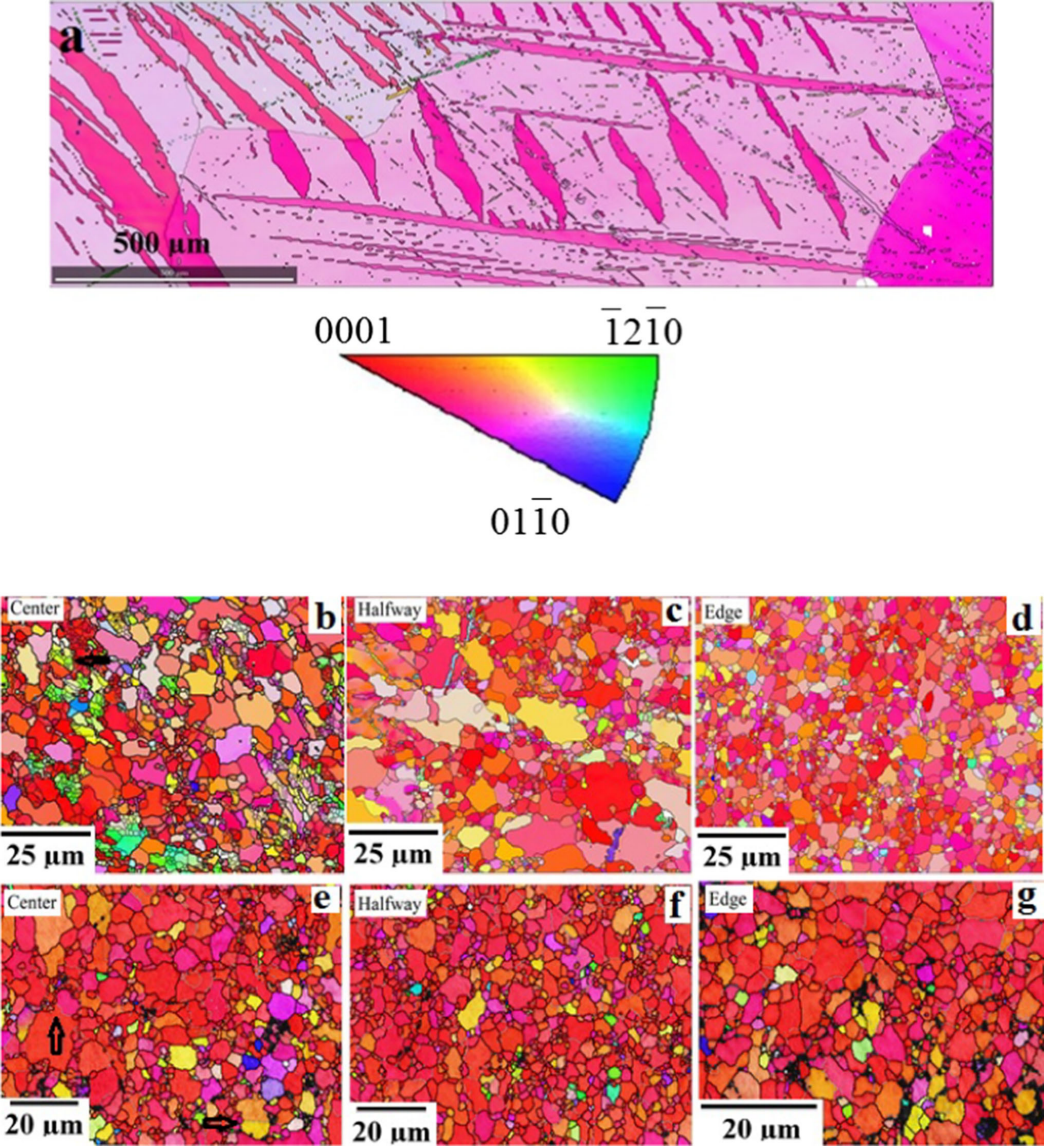
EBSD images of the surface in **a** cast Mg and in HPT-processed Mg with **b**–**d** 1 and **e**–**g** 5 turns; the positions are **b**, **e** center, **c**, **f** halfway and **d**, **g** edge of the samples; high-angle grain boundaries are black and low-angle boundaries are gray in color with black spots denoting non-indexed pixels

The microstructures of the top surfaces in the cast and HPT-processed Mg samples after one and five turns are shown in the EBSD overlapped inverse pole figure (IPF) and grain boundary maps in Fig. 2 where the colors denote different grain orientations as depicted in the upper unit triangle. It can be seen that the cast Mg in Fig. 2a has a very coarse grain structure of a few millimeters with some twins whereas the grain sizes in the HPT-processed samples were refined significantly to ~ 1.6–4.0 µm in Fig. 2b–g for the centers, halfway positions and edges of the disks, respectively. A comprehensive summary is given in Table 1 of the grain sizes at the top surfaces for these three positions for both a combination of low-angle grain boundaries (LAGBs having misorientations below 15°) and high-angle grain boundaries (HAGBs with misorientations at and above 15°) and for the HAGBs only.

**Table 1:** Average grain size values (µm) with the standard deviation at different locations in the HPT-processed Mg

Location	*N* = 1		*N* = 5	
	LAGB + HAGB	HAGB	LAGB + HAGB	HAGB
Center	3.8 ± 2.6	4.1 ± 4.6	2.0 ± 1.7	2.2 ± 1.9
Halfway	1.8 ± 1.5	2.4 ± 2	2.0 ± 1.3	2.2 ± 1.6
Edge	1.6 ± 0.4	1.6 ± 0.2	1.6 ± 1.2	1.8 ± 1.4

*N* number of turns, *LAGB* low-angle grain boundary (misorientation < 15°), *HAGB* high-angle grain boundary (misorientation > 15°)

Figure 2b–g relates to the surface microstructures of the HPT-processed disks at these different radial locations within the circular samples and inspection suggests that they exhibit typical bimodal grain size distributions. The microstructures of both samples at the center after one and five turns in Fig. 2b, e are composed of a relatively large fraction of coarser grains mixed together with a small fraction of fine grains. By contrast, the fractions and sizes of the coarser grains are reduced at greater distances from the center. These results demonstrate, therefore, that the grain structure tends to become more uniform from the center to the edge along the radial direction and this is reasonable considering the increase in strain with increasing distance from the center.

According to Fig. 2, the HPT processing refined the grain structure and resulted in much refined equiaxed grains. It can be seen that, in addition to grains of several micrometers in size, there are much smaller grains of a few micrometers. This is most evident after one turn and at the center and therefore after the smallest strain (Fig. 2b) where very small grains are located in groups which are evidently within prior grains and they are not formed as necklace-like structures along the original grain boundaries of the large grains. None of these grain groups are red so that they are without the basal texture component. All very small grains have the same orientation, so that the grain boundaries between them are LAGBs and they correspond to subgrain boundaries. It is concluded that these grains are not recrystallized but they are dynamically recovered. Because the groups are absent after higher strains (from Fig. 2c onwards), although smaller and larger grains remain evident in the images, it seems that continuous dynamic recrystallization takes place in these grain areas at larger strains where the large grains are a consequence of discontinuous recrystallization.

A bimodal grain structure was found that was similar to that reported after HPT of Mg in earlier reports [15, 20]. For example, there was a bimodal microstructure including large recrystallized grains, free of dislocations, in the steady-state condition with an average grain size of about 1 μm [15] and there is another report of equiaxed grains with a size of about 1–5 μm [20]. Because the latter grain size increased with increased applied pressure, it was concluded that the grains are formed by both discontinuous static and dynamic recrystallization mechanisms. Thus, after 5 turns and 6 GPa pressure two different types of regions were reported, with “deformed regions” having ultrafine grains and a high density of dislocations and “recrystallized regions” with substantially larger grains (1–5 μm) and almost free of dislocations [21]. Thus, the bimodal grain structure observed in the present investigation has similarities to several earlier reports.

The sample processed by five turns also demonstrated a similar variation in the grain structure as in the one-turn sample. Furthermore, the grain size showed no significant change with increasing numbers of turns as documented in Table 1 except only at the disk center where there is no torsional straining and consequently there was only a high compressive load.

**Figure 3:**
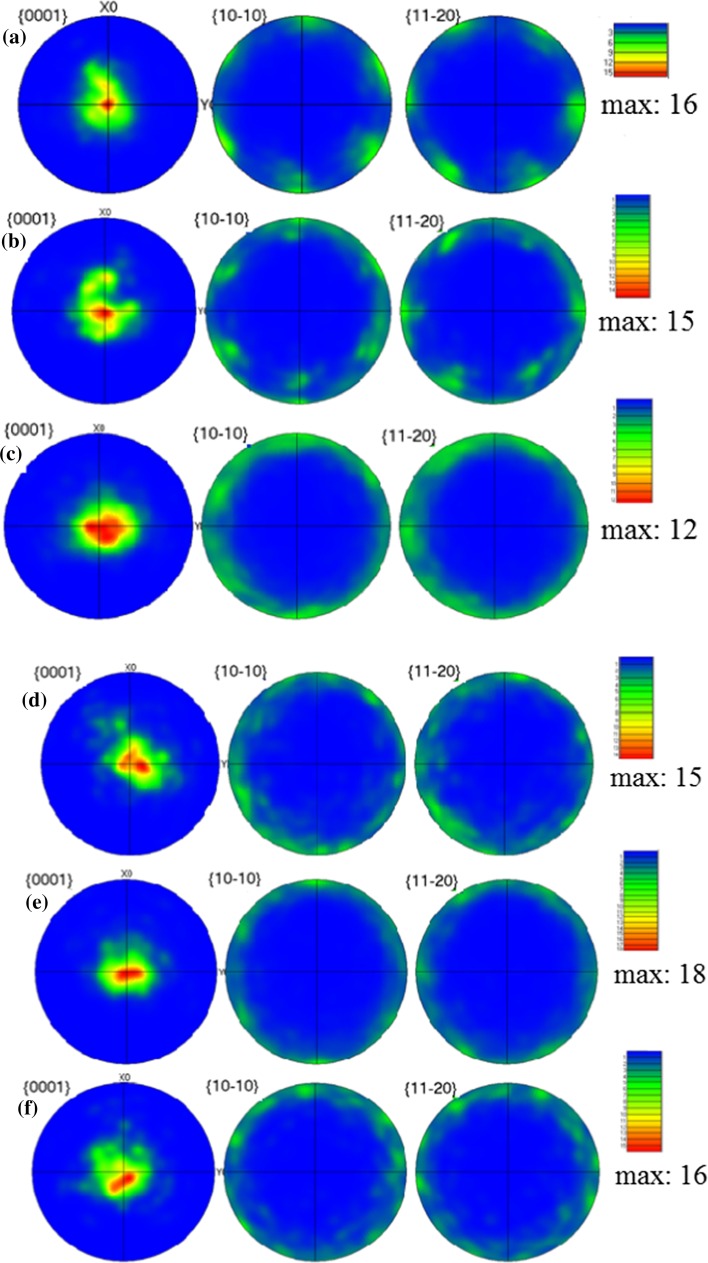
Pole figures of the surface of the HPT-processed samples at different locations: **a**–**c** 1 turn, **d**–**f** 5 turns with positions of **a**, **d** center, **b**, **e** halfway and **c**, **f** edge

**Figure 4:**
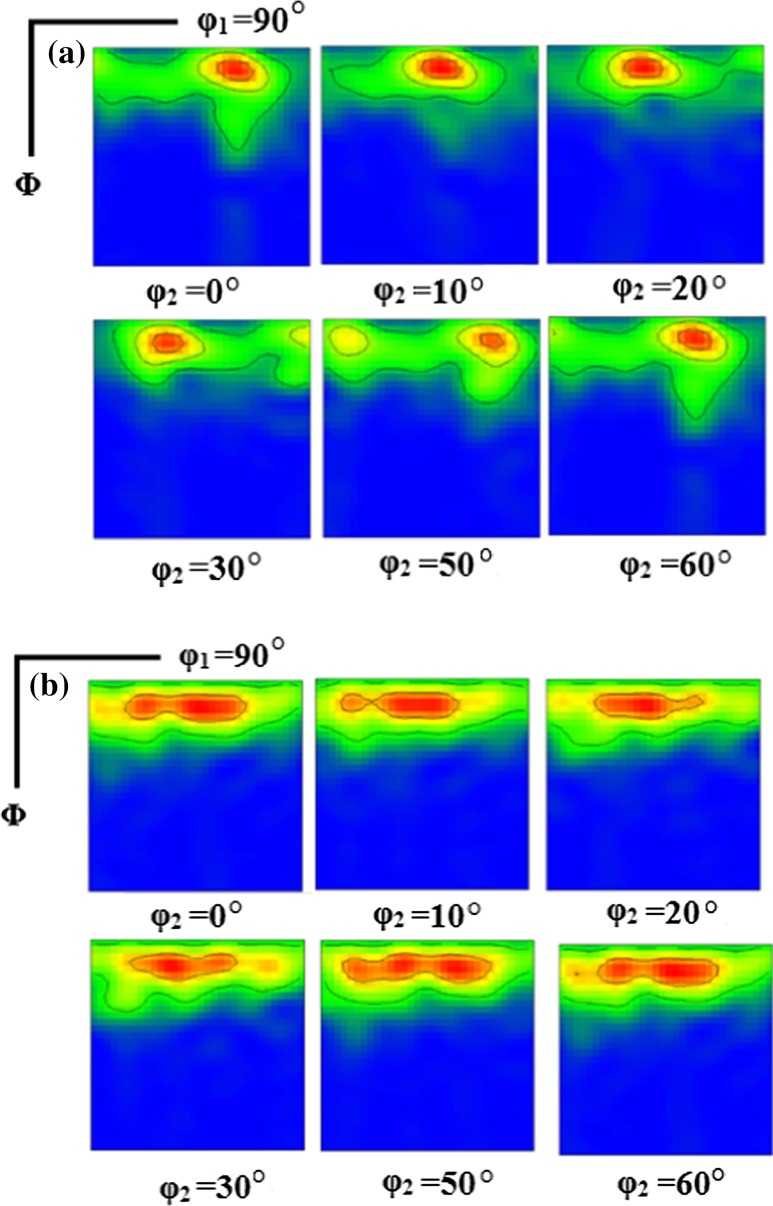
ODF plots for surfaces of an HPT sample with 1 turn at **a** center and **b** edge

In the EBSD images in Fig. 2, it is readily apparent that most of the grains are red in color. This means that the {0001} planes are parallel to the disk surface and this preferred orientation is essentially maintained over the entire surface. Figure 3 displays the pole figures of the HPT-processed Mg at different locations after (a–c) 1 and (d–f) 5 turns. Thus, the pole figures after one turn at the center of the sample demonstrate a {0001} <10$\bar{1}$0> basal texture with an increasing intensity with increasing strain toward the edge of the sample. The ODF plots in Fig. 4 also confirm the texture evolution on the surface through the radius of the sample and by increasing to five turns of HPT a homogenous {0001} basal texture exists over the whole sample surface as is evident in Fig. 3. Since the as-cast Mg had coarse grains, and in the cross sections only two or three grains were visible, it was not possible to determine the texture.

**Figure 5:**
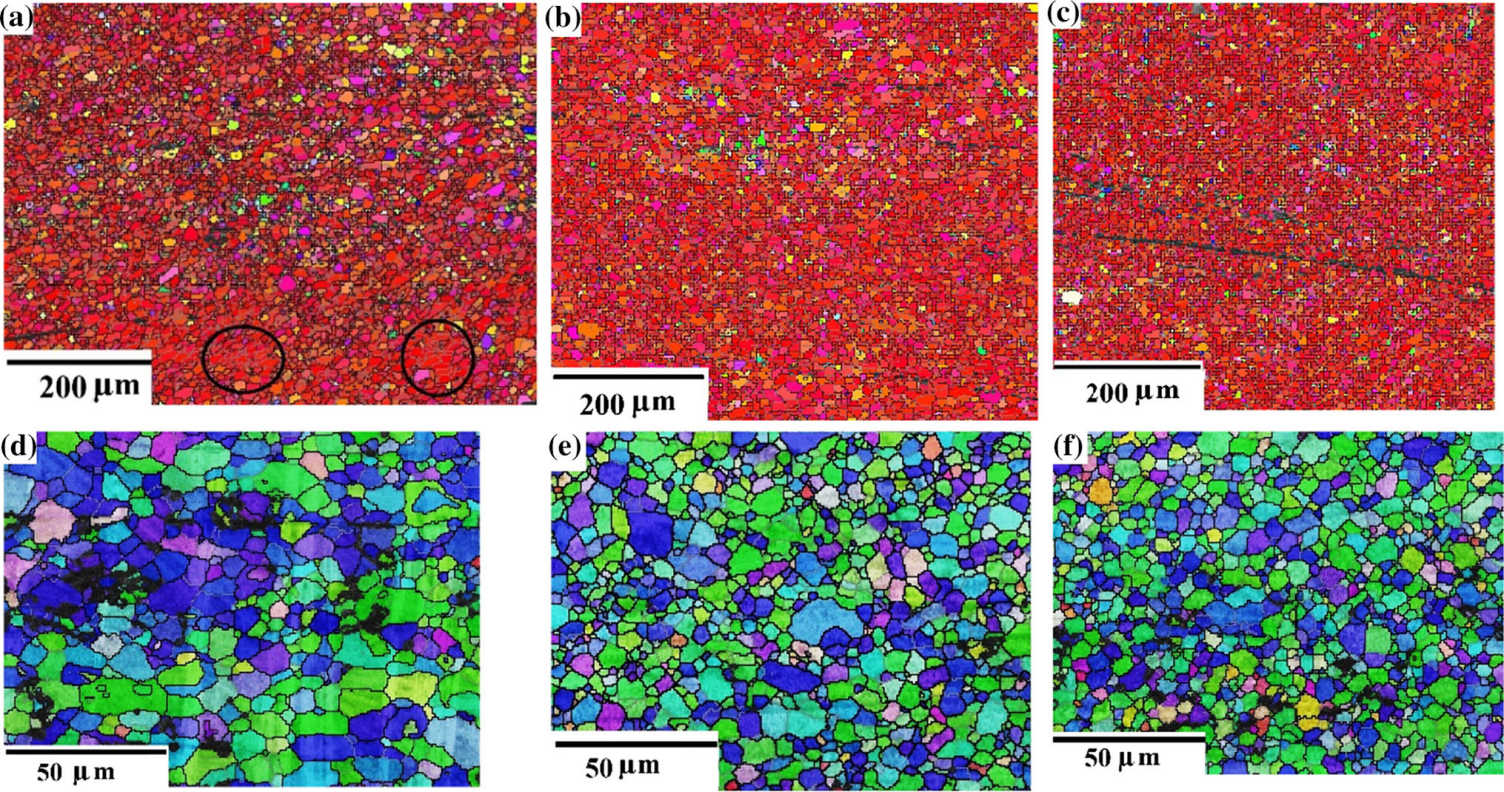
EBSD images over the cross sections of an HPT-processed Mg sample after one turn. **a**–**c** Images from the top to the bottom, the view direction toward the upper surface. **d**–**f** High magnification images, the view direction toward the normal of the cross-sectional surface. **a**, **d** Center, **b**, **e** halfway, **c**, **f** edge. High-angle grain boundaries are black and low-angle boundaries gray in color. Two large recovered grains are encircled in **a**

In order to check the homogeneity of the grain structure through the cross sections of the HPT-processed disks, the microstructure over the whole thickness after one turn is presented in Fig. 5 in three radial locations. The orientation of the grains is viewed in two directions, the direction perpendicular to the disk surface (Fig. 5a–c) as well as in a direction parallel to the surface (Fig. 5d–f). The first images show red grains so that there is a strong basal texture through the disk parallel to the surface. The final three images (Fig. 5d–f) show bluish and greenish colors so that most of the grains have orientations close to (01$\bar{1}$0) and ($\bar{1}$2$\bar{1}$0) when looking toward the cross section of one-half of the disk. The microstructure through the thickness is not entirely homogeneous after one turn in the center region and there are a few flattened (about 100 μm long) grains containing LAGBs which indicate the occurrence of recovery in this region (see Fig. 5a, the encircled larger grains). Very small recovered grains of a few micrometers in size were also observed at the surface layer (in Fig. 2b) but due to a much lower resolution in Fig. 5a these grains cannot be resolved. It appears that dynamic recrystallization can occur in most grains already during one turn but also there are areas of dynamic recovery. Inhomogeneities through the cross sections of HPT-processed disks were also reported in some other investigations [22–24]. It is noted that in the halfway and edge regions in Fig. 5b, c the grains appear to be more uniform in size and recrystallized, which is similar to the conclusions from Fig. 2c, d.

**Figure 6:**
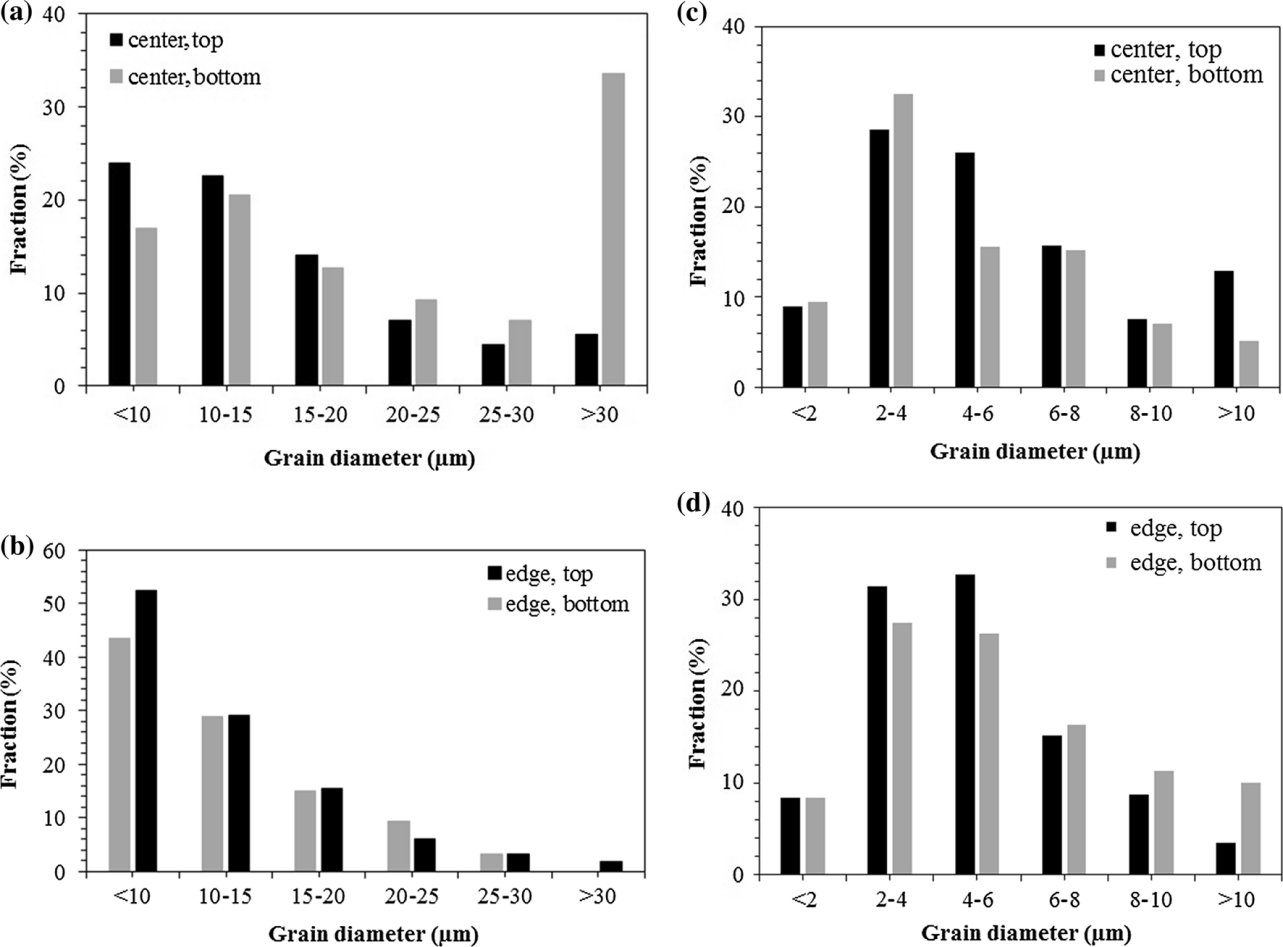
Grain size distributions in cross sections of HPT samples: **a**, **b** 1 turn and **c**, **d** 5 turns with positions of **a**, **c** center, **b**, **d** edge for top and bottom measurements

Figure 6 presents pictorial displays of the distributions of the area fractions of different grain sizes at the center and edge for both (a and b) one and (c and d) five turns. After one turn at the center in Fig. 6a, there is a distinct difference between the top and bottom layer, with the former having a higher fraction of fine (< 10 µm) grains and the latter having more numerous larger (> 30 µm) grains. This difference is not so pronounced at the edge in Fig. 6b and after five turns the grain sizes became more homogeneous in Fig. 6c–d and the grain size distributions through the thickness after 5 turns are similar in the top and bottom of the sample.

### Microhardness

**Figure 7:**
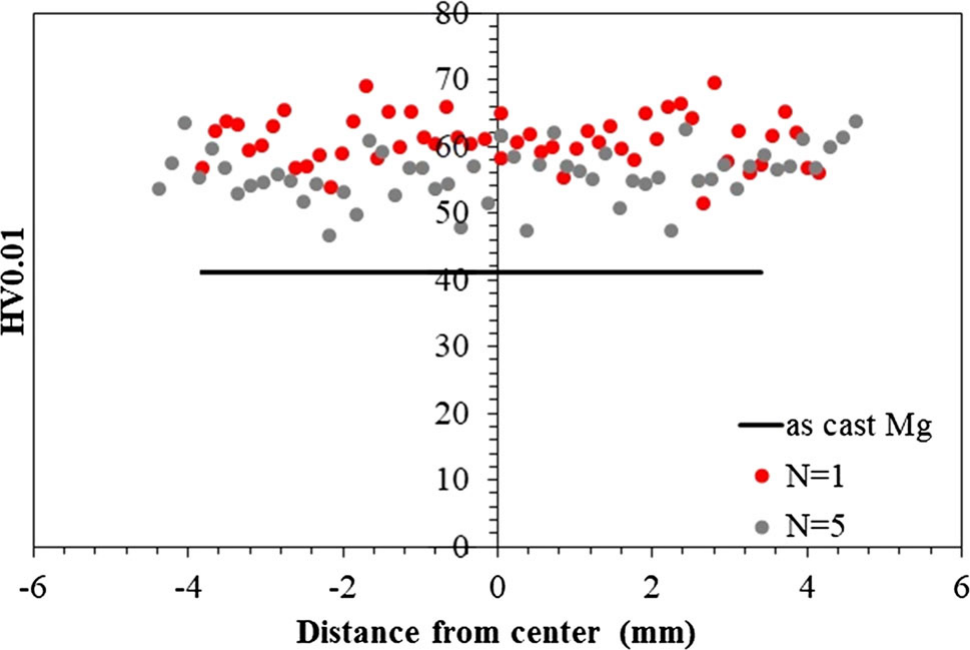
Microhardness values of the as-cast and HPT-processed samples versus the distance from the center of the samples

The microhardness of the samples was measured in order to estimate the changes in the microstructure and strength attributed to the HPT processing. Figure 7 shows the variation in Vickers microhardness as a function of distance from the center of the disk samples in the as-cast material and after 1 and 5 turns. The initial hardness of the as-cast Mg was relatively high, 41 Hv_0.01_. The results show that the hardness increases clearly by one turn of HPT processing to about 60 Hv_0.01_. However, increasing the turns to 5 slightly decreases the hardness. Thus, the hardness variation is irregular and any sign of a peak or any trend by distance from the centers of the disks is not generally apparent from these data. These results are in agreement with other observations [15, 25]. For example, there is a report that even after less than one turn there was an increase in hardness with respect to the distance from the center but at higher deformations the opposite occurred due to dynamic softening.

In practice, the hardness is affected by three separate factors: the finer grain size, the texture of the HPT samples and the higher dislocation density which is an inherent feature of the SPD-processed microstructure. In this study, the microhardness of HPT samples did not change by increasing the distance from the center although the grain size was slightly reduced. The basal texture is quite similar over the total surface as shown in Fig. 2, 3 and 4. Therefore, the dislocation density and thus the extent of recovery must vary with strain and have the main impact on the microhardness values. After one turn at the center, there are numerous subgrains and it is concluded that there is a higher dislocation density in an incompletely recrystallized structure which accounts for the higher hardness in this stage.

**Figure 8:**
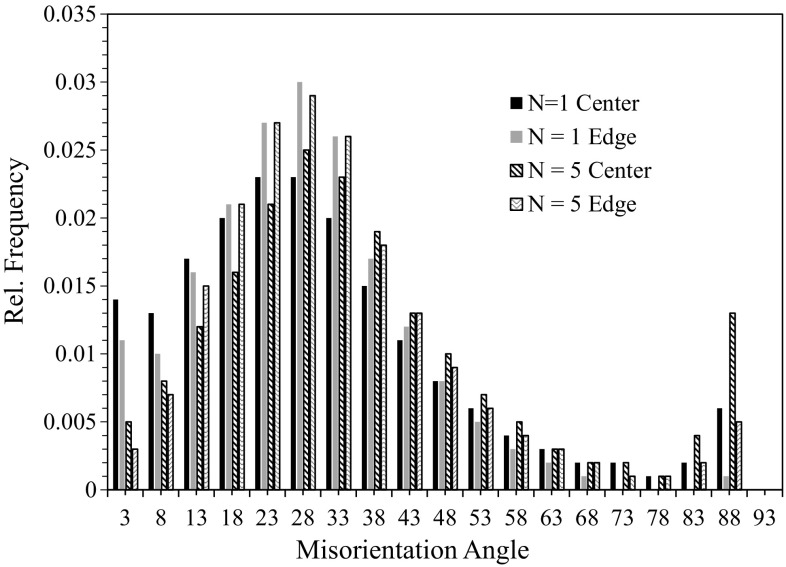
Misorientation angles in HPT samples at center and edge after 1 and 5 turns

The grain boundary misorientation distributions displayed in Fig. 8 also confirm the existence of higher numbers of LAGBs, and therefore of recovered grains, after 1 turn compared with 5 turns, where it was shown earlier that the two main peaks, appearing at about 27° and 88°, correspond to the existence of doubled and tensile twins [26]. The fraction of LAGBs varies along the radial directions due to the different strain concentrations and in practice the fraction is higher at the center than in the edge region. Thus, after 5 turns a fully recrystallized structure was achieved with a lower hardness instead of a partly recovered structure.

It has been noted that a softening behavior tends to occur in pure metals when the homologous temperature is larger than ~ 0.3 [27] and in the present investigation the homologous temperature was ~ 0.32. It was observed by transmission electron microscopy that a replacement of the “deformed regions” in HPT Mg by dislocation-free recrystallized grains led to a softening [21] and it was suggested that, due to the faster defect recovery in Mg compared to metals such as Al, Cu and Ag, a saturation in hardening may occur in Mg at lower strains than in those other metals [25].

It was also noted earlier that additional HPT processing after 1/8 turn produced no significant increase in the microhardness [25] and this hardening at very small strains is probably due to the hexagonal close-packed (hcp) structure which provides basal slip and only two independent slip systems which are easily activated at room temperature. In order to fulfill the von Mises criterion, twinning and/or non-basal slip is also required at the onset of the HPT deformation [28].

### Tensile properties

**Figure 9:**
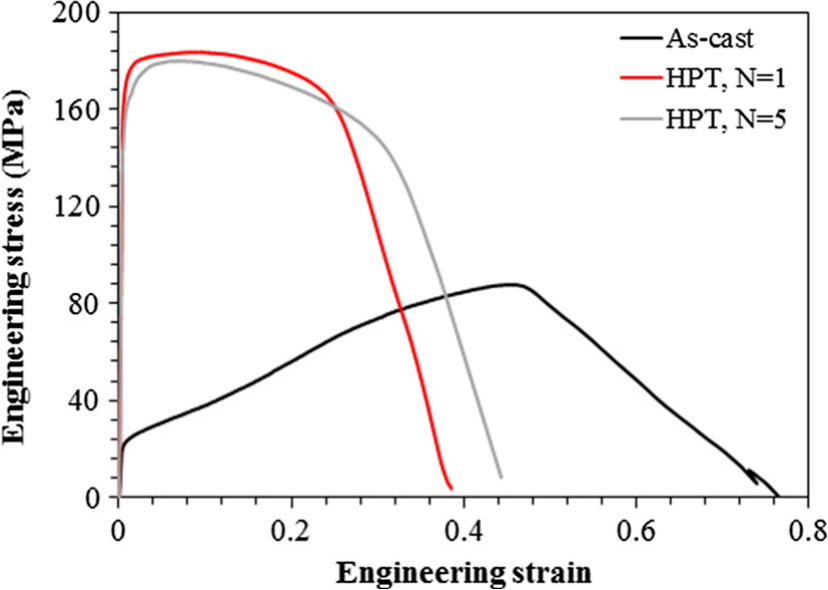
Engineering stress–strain curves of the cast and HPT-processed samples at room temperature

Processing by SPD procedures is used conventionally to improve the strength properties of bulk solids. Accordingly, tensile testing was conducted both before and after HPT processing and representative engineering stress–strain curves for the cast and HPT-processed miniature samples are plotted in Fig. 9. These curves show that the yield and ultimate tensile strengths are markedly increased by HPT processing as listed in Table 2, but conversely, and as anticipated from the paradox of strength and ductility [29], the overall elongations are reduced through HPT processing. The concave tensile behavior of the cast Mg was explained previously [26]. Apart from the grain refinement and the dislocation density, texture strengthening also contributes to strength of Mg. In practice, texture strengthening is generally caused by deformation along the forming direction which limits basal slip and is beneficial for the activation of non-basal slip and thereby results in an increasing yield strength. It should also be considered that the low stacking fault energy on the basal plane (36 mJ m^−2^) may reduce the dislocation slip and cause strain hardening [30].

**Table 2:** Tensile properties of as-cast and HPT-processed Mg

Sample	Yield strength (MPa)	Tensile strength (MPa)	Elongation (%)
As-cast	18 ± 0.4	83 ± 1.2	46
*N* = 1	150 ± 9.2	181 ± 0.7	30
*N* = 5	142 ± 5.6	180 ± 7.1	38

### Corrosion resistance

Corrosion resistance is one of the most important properties for biomedical applications since corrosion products may give some cytotoxicity effects and low corrosion resistance can cause a significant reduction in mechanical strength. Therefore, in this research the corrosion resistance of the samples was examined to determine whether HPT retards or facilitates the corrosion of Mg.

**Figure 10:**
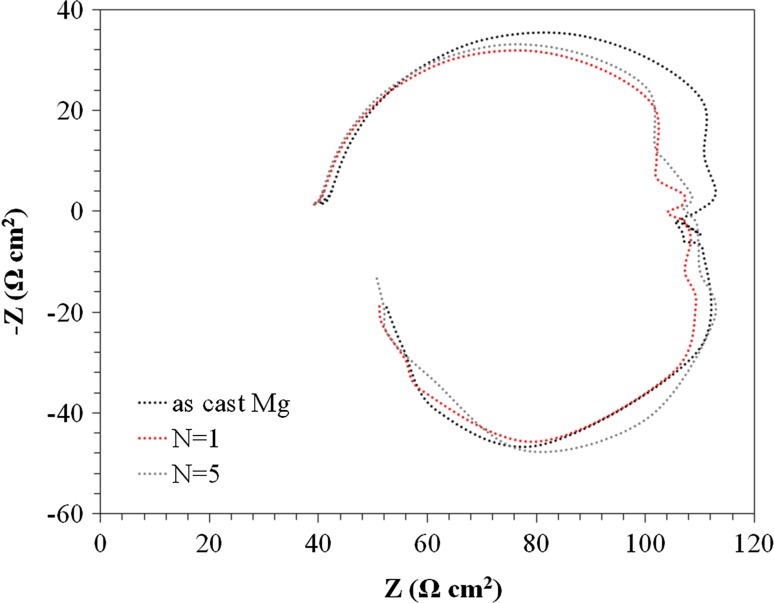
Nyquist plots of the cast and HPT-processed Mg in an NaCl solution

**Figure 11:**
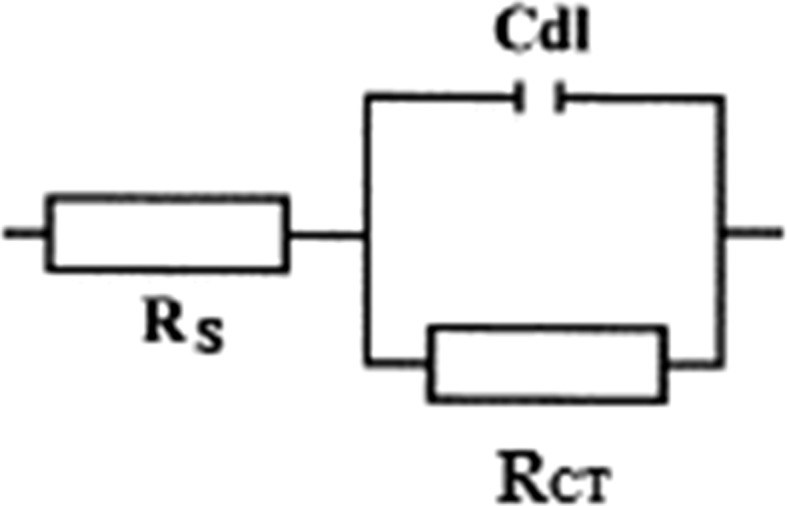
Equivalent circuits used for fitting the impedance data

Figure 10 shows the Nyquist impedance plots for the cast and HPT-processed Mg when using NaCl solution. It can be seen that the plots for all samples include one capacitive loop and one inductive loop at low frequency. The capacitive loop has been assigned to charge transfer and film effects and the low-frequency inductive loop is related to the relaxation of adsorbed species such as Mg^+^ and non-stationary conditions [31]. The same shapes of the Nyquist plots, as well as the same diameters of the capacitive loops for all samples, suggest a similar corrosion mechanism and equal corrosion rates. Two earlier studies determined two time constants for Mg and its alloys immersed in NaCl [31, 32] where a reduction in the corrosion resistance of pure Mg was observed after processing by ECAP. Figure 11 shows the equivalent circuit that was used to fit the impedance data by ZSimpwin software and the results are listed in Table 3 where *R*_s_ is the electrolyte resistance between the working and reference electrode, *C*_dl_ is the electrochemical double-layer capacitance at the substrate/electrolyte interface and *R*_CT_ is the charge transfer resistance. For a best fit with the experimental data, the inductive part was omitted from the equivalent circuit and the fitting was carried out between 1 and 100 kHz.

**Table 3:** Fitting data related to the impedance measurements for as-cast and HPT-processed Mg

Parameters	As-cast Mg	N = 1	N = 5
*R*_s_ (Ω cm^2^)	42	40	40
*C*_dl_ (μF cm^−2^)	4.1 × 10^−5^	4.7 × 10^−5^	5.3 × 10^−5^
*R*_CT_ (Ω cm^2^)	71	65	67

## Discussion

The results from this investigation are generally consistent with expectations. Processing by HPT produces a very significant grain refinement in the cast pure Mg with a grain size reduction at the edge of the disk from an initial value of several millimeters to processed values of ~ 1.6 μm after both 1 and 5 turns. Generally, processing of pure Mg is difficult using SPD techniques. For example, the processing of pure Mg by the alternative procedure of ECAP at 673 K led to a reduction in grain size from ~ 400 μm to a grain size larger than 100 μm after processing through 2 passes [33]. This difficulty arose because of the lack of slip systems in the hcp structure and the potential for introducing cracking or large-scale segmentation [34, 35]. The problem was at least partially alleviated when using ECAP by introducing an initial extrusion step and then processing at an elevated temperature [36]. In the present investigation, the HPT was conducted directly using the cast Mg without any extrusion and the processing was performed at room temperature where the high hydrostatic pressure inherent in HPT permitted successful processing of the pure metal without the introduction of any cracking. Using room temperature for the processing operation is advantageous because it avoids the occurrence of significant grain growth which will occur when conducting the HPT at elevated temperatures [12].

The hardness behavior in these experiments is unusual because in many materials, including Mg alloys [37], the hardness increases with increasing numbers of turns and then saturates after about 5 to 10 turns [27, 38, 39]. In the present experiments, the hardness increased from the cast condition but it was slightly higher after 1 turn than after 5 turns due to the advent of recrystallization.

According to the present data, there is no significant improvement in the corrosion resistance by applying the HPT processing to pure cast Mg. Nevertheless, it was shown earlier that HPT-treated samples possessed a lower biodegradation rate with respect to the as-cast Mg alloy due to the relieving of internal stresses [40]. In the case of Mg, the oxide layer is crystalline in nature and the microstructure is discontinuous between the oxide layer and the matrix leading to a high compressive stress within this layer. It was noted also that the increase in grain boundary density can reduce the mismatch and disorder between the Mg surface and the oxide layer [32] and, in addition, it has been confirmed that a surface with an (0001) plane has improved corrosion resistance by comparison with (10$\bar{1}$0) and (11$\bar{2}$0) planes even when the grain size remains the same [41]. An earlier study on pure Mg suggested that processing by ECAP or HPT leads to an overall change in the macroscopic corrosion behavior so that, whereas pure Mg generally exhibits a localized corrosion in the as-cast condition, processing by HPT produces a reasonably homogeneous corrosion surface [40].

The apparent general lack of any major improvement in the corrosion behavior in this research is consistent with results on some other materials such as commercial purity Ti processed by HPT [42]. Nevertheless, based on the results in Table 3, it appears that other metallurgical and structural factors associated with HPT processing, such as the presence of residual stresses or the introduction of high dislocation densities, may have a larger influence on the corrosion behavior than either the grain size or the texture. Accordingly, it is recommended that additional experiments are conducted to more fully characterize the corrosion resistance of pure Mg after processing by HPT.

## Summary and conclusions

1. Cast pure Mg disks were processed by HPT at room temperature using 1 or 5 turns. The results show that the processing refines the grain size from a few millimeters to a few micrometers and also creates a strong dominant basal texture through the disk.
2. Measurements showed that the grain size was effectively refined and the basal texture was intense even after processing through only 1 turn. Nevertheless, the microstructure became more homogeneous throughout the disks by increasing the HPT processing to 5 turns. The refined grain structure also exhibited recovered grains with low-angle grain boundaries at the surface layer and a few large recovered grains at the bottom layer of the disk in the center region after one turn.
3. The highly refined grain size, substructure within a few grains and the basal texture resulted in a slightly higher microhardness after 1 turn than after 5 turns. The yield stress was also enhanced to about seven times in comparison with the cast Mg.
4. The results suggest that the corrosion resistance was not significantly improved by using HPT processing but more experiments are needed to more fully characterize the corrosion resistance.
